# Mono-Energy Coronary Angiography with a Compact Synchrotron Source

**DOI:** 10.1038/srep42211

**Published:** 2017-02-09

**Authors:** Elena Eggl, Korbinian Mechlem, Eva Braig, Stephanie Kulpe, Martin Dierolf, Benedikt Günther, Klaus Achterhold, Julia Herzen, Bernhard Gleich, Ernst Rummeny, Peter B. Noёl, Franz Pfeiffer, Daniela Muenzel

**Affiliations:** 1 Chair of Biomedical Physics, Department of Physics, Technical University of Munich, James-Franck-Straße 1, 85748 Garching, Germany; 2 Munich School of Bioengineering, Technical University of Munich, Boltzmannstraße 11, 85748 Garching, Germany; 3 Department of Diagnostic and Interventional Radiology, Klinikum rechts der Isar, Technical University of Munich, Ismaninger Straße 22, 81675 München, Germany; 4 Max-Planck-Institut für Quantenoptik, Hans-Kopfermann-Straße 1, 85748 Garching, Germany

## Abstract

X-ray coronary angiography is an invaluable tool for the diagnosis of coronary artery disease. However, the use of iodine-based contrast media can be contraindicated for patients who present with chronic renal insufficiency or with severe iodine allergy. These patients could benefit from a reduced contrast agent concentration, possibly achieved through application of a mono-energetic x-ray beam. While large-scale synchrotrons are impractical for daily clinical use, the technology of compact synchrotron sources strongly advanced during the last decade. Here we present a quantitative analysis of the benefits a compact synchrotron source can offer in coronary angiography. Simulated projection data from quasi-mono-energetic and conventional x-ray tube spectra is used for a CNR comparison. Results show that compact synchrotron spectra would allow for a significant reduction of contrast media. Experimentally, we demonstrate the feasibility of coronary angiography at the Munich Compact Light Source, the first commercial installation of a compact synchrotron source.

X-ray imaging is an invaluable diagnostic tool in clinical practice. While conventional x-ray tubes are well established and reliable in clinical x-ray imaging, their broad bremsstrahlung spectra impose a number of drawbacks with respect to image quality. The polychromatic spectrum can introduce beam hardening artifacts or impede the aim to optimize both the dose level and the image quality, as the x-ray energy cannot be tuned directly to the desired range. On the other hand, 3rd generation synchrotron sources offer highly brilliant and monochromatic x-ray beams, but their high spatial and financial demands make their clinical use impracticable. To close this performance gap, new types of x-ray sources have been investigated throughout the last decades. One possibility are compact light sources (CLS) based on inverse Compton scattering^1^, which provide a quasi-mono-energetic, tunable and partially coherent x-ray beam. The first commercially sold system has only recently been installed in Munich, Germany^2^.

One of the clinical applications that could benefit significantly from a mono-energetic x-ray beam is coronary angiography, as it relies on contrast media application. A mono-energetic x-ray beam could thoroughly exploit the sudden increase of the absorption coefficient of a contrast medium at its K-edge.

Coronary angiography is an invaluable tool for the diagnosis of coronary disease. Complications are frequently associated with the high amount of iodine-based contrast media that are injected during the catheterization procedure. Especially for patients presenting with pre-existing renal insufficiency, there is a high risk to suffer from renal failure due to nephrotoxic effects of iodine-based contrast agents, resulting in severe renal dysfunction and a high risk of subsequent dialysis treatment^3^,^4^,^5^,^6^. In addition, several patients show allergic reactions following iodine injection, with the life-threatening risk of anaphylaxis^6^. A high amount of iodine can also induce hyperthyroidism by influencing the endocrine function of the thyroid gland^7^. The number of patients harmed by these adverse effects can be lowered if a good diagnostic image quality is achieved with a reduced amount of contrast media being injected.

Research efforts have furthermore been made to examine the use of gadolinium-based contrast media for coronary angiography, as gadolinium is a frequently used contrast agent for MRI imaging and is also successfully used for cardiovascular MR^8^. Even though there are some drawbacks related to the use of gadolinium for x-ray coronary angiography, such as a reduced image quality due to the lower possible concentration, several studies have shown the feasibility of applying gadolinium for patients with e.g. severe iodine allergy^9^,^10^,^11^,^12^,^13^,^14^. Implementing a compact synchrotron source with an x-ray energy tuned directly above the gadolinium absorption edge could help to improve the image quality in the future.

Here we analyze the quantitative effect of quasi-mono-energetic x-ray spectra on the contrast-to-noise ratio (CNR) of coronary angiography. We used virtual projection images calculated for a segmented human coronary artery from real patient data for a comparison of typically used clinical x-ray spectra with compact synchrotron spectra. For an iodine-based contrast agent, we compare a conventional x-ray tube spectrum at 60 kVp (peak kilovoltage) with a quasi-mono-energetic spectrum at 35 keV. A 90 kVp conventional x-ray tube spectrum and a 55 keV quasi-mono-energetic spectrum are examined for the application of gadolinium-based contrast media. Finally, we present experimental coronary angiography data acquired at the Munich Compact Light Source (MuCLS) of a porcine heart using iodine-based contrast media at an x-ray energy of 35 keV.

## Results

### Simulated angiography images for iodine-based contrast media

**Figure 1:**
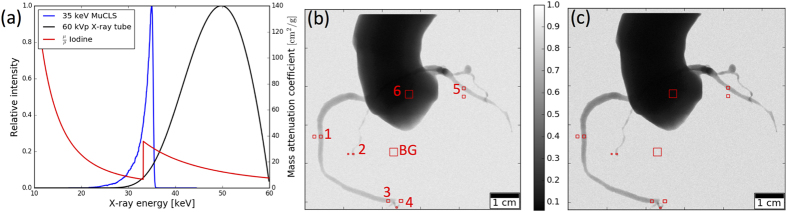
(**a**) Measured MuCLS spectrum at 35 keV peak energy, x-ray tube spectrum at 60 kVp and mass attenuation coefficient of iodine. (**b**) Simulated iodine-based angiography image for the 60 kVp x-ray tube spectrum. (**c**) Simulated iodine-based angiography image for the 35 keV MuCLS spectrum.

X-ray projection images of a human coronary artery filled with iodine-based contrast media were simulated for a conventional x-ray tube spectrum at 60 kVp (cf. Fig. 1(b)) and a compact synchrotron source spectrum at 35 keV (Fig. 1(c)). Visual comparison of the two images shows better visibility of small vessels for the quasi-mono-energetic image (c). The coronary artery tree is clearly visible with the right coronary artery (ROI 1) and the left coronary artery with its main branch, the left anterior descending artery (5). In addition, a small arterial branch arising from the aorta can be visualized (2). The analysis of the CNR presented in Table 1 supports this impression. Except for ROI 6 located in the aorta, where the CNR is lower for the MuCLS spectrum due to photon starvation, the quasi-mono-energetic spectrum yields CNR values which lie 17–22% above those for the conventional x-ray tube spectrum. When lowering the iodine concentration by 1/3, the advantage of the MuCLS spectrum increases to 24–28%. The CNR values of the quasi-mono-energetic spectrum at lower iodine concentration are comparable with those of the conventional spectrum at a 50% higher iodine concentration.

### Simulated angiography images for gadolinium-based contrast media

**Figure 2:**
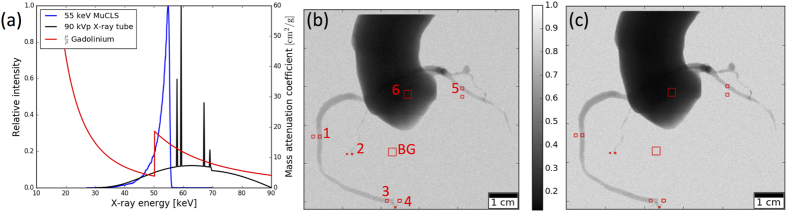
(**a**) MuCLS spectrum rescaled at 55.8 keV peak energy, x-ray tube spectrum at 90 kVp and mass attenuation coefficient of gadolinium. (**b**) Simulated gadolinium-based angiography image for the 90 kVp x-ray tube spectrum. (**c**) Simulated gadolinium-based angiography image for the 55 keV MuCLS spectrum.

Simulated x-ray projection images obtained for gadolinium-based contrast agent with a 90 kVp conventional x-ray tube spectrum and a 55 keV CLS spectrum are displayed in Fig. 2(b,c), respectively. The CLS spectrum provides a superior perceptability of the smaller vessels. The quantitative evaluation of the CNR is presented in Table 2. The CNR achieved with a quasi-mono-energetic MuCLS spectrum is 41–62% higher than with an x-ray tube spectrum in ROIs 1–5, and 12% higher in the aorta-ROI (6). For a reduced gadolinium concentration, the improvement with a mono-energetic spectrum even increases for most ROIs and is 30–51% higher compared to a conventional spectrum. As in the previous section, CNR values achieved with the MuCLS spectrum at lower gadolinium concentration are similar to those from the conventional spectrum at a 50% higher concentration.

### Angiography image acquired at MuCLS

**Figure 3:**
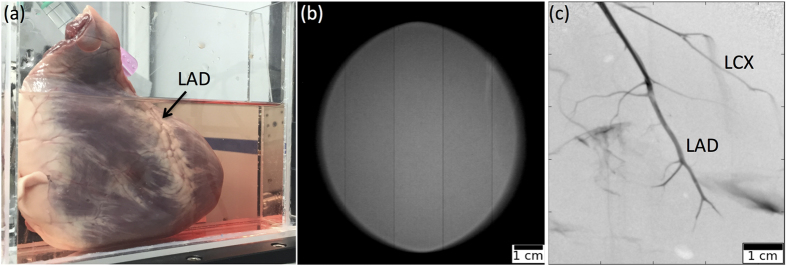
MuCLS angiography image. (**a**) Photograph of the sample in waterbath. (**b**) Empty image of full MuCLS beam. (**c**) Quasi-mono-energetic angiography image of a porcine heart acquired at the MuCLS, with iodine-based contrast agent injected into the left coronary artery. Visible are the left anterior descending artery (LAD) and the left circumflex artery (LCX).

The quasi-mono-energetic angiography image of a porcine heart acquired at the MuCLS is presented in Fig. 3(c). The left anterior descending artery (LAD) and the left circumflex artery (LCX) arising from the left main coronary artery (not shown) are imaged with an excellent delineation even of the small side branches. Although the image quality is compromised by some contrast agent spread outside of the vessels, even small vessels are easily recognized. The background of the image is flat due to the waterbath in which the specimen was placed.

## Discussion

By numerical simulations and experimental images, we showed that a quasi-mono-energetic spectrum produced by an inverse Compton x-ray source is beneficial for coronary angiography. The feasibility of mono-energy angiography at the MuCLS was experimentally demonstrated. In the future, the experimental approach can be extended to dual-energy K-edge subtraction angiography, where the beam energy is oscillated rapidly between above and below the edge. Technically, this energy change could either be realized through the electron beam energy or the laser wavelength (eq. 2). If the electron beam energy should be altered, the energy of the injected and stored electron beam could be oscillated at the few Hz timescale using laminated magnets. Alternatively, laser beams of two different wavelengths could be stored in the same optical cavity.

A simulation based on a polychromatic forward model allowed for a quantitative analysis of the CNR achieved for a segmented coronary artery for such a mono-energetic spectrum in comparison with a conventional x-ray tube spectrum. A quasi-mono-energetic spectrum tuned directly above the K-edge of iodine yielded higher CNR than a conventional x-ray tube spectrum at 60 kVp as typically used for coronary angiography. A mono-energetic spectrum would allow for a reduction of the iodine concentration by approximately 20–30% at almost equal CNR, which can facilitate the administration of the contrast agent for the patients. However, due to penetration issues and dose constraints, a 35 keV MuCLS spectrum is mainly restricted to small-animal imaging. The application of a the MuCLS for coronary angiography could profit from a better suited detector, e.g. a single photon counting detector. A flatpanel detector with CsI sensor was chosen for the simulation as it is widely used for clinical coronary angiography.

For contrast media based on gadolinium, the CNR values are in general lower than for iodine, on the one hand due to the higher sample thickness and on the other hand due to the lower absorption coefficient of gadolinium. Importantly, the improvement of the CNR achieved with a compact synchrotron source tuned directly above the K-edge compared to a conventional spectrum at 90 kVp is even higher than for the iodine case. An increase in CNR of over 40% was observed for the MuCLS spectrum. Gadolinium is a well established contrast agent in magnetic resonance imaging and has also been investigated for x-ray imaging. Since the allowed dosage of gadolinium is limited^13^, the possibility of reducing the gadolinium concentration by 1/3 at equal CNR for a quasi-mono-energetic spectrum compared to an x-ray tube spectrum motivates the implementation of compact synchrotron sources. Especially patients with severe iodine allergy or chronic renal insufficiency could profit from using gadolinium-based contrast agent in combination with a CLS for coronary angiography.

While the MuCLS is limited to a maximum x-ray energy of 35 keV, the field of compact synchrotron sources is strongly under research^2^,^15^,^16^ and an extension to higher x-ray energies above 50 keV appears feasible. Higher electron and laser photon energies will require an adaptation of electron storage ring and laser cavity design. Furthermore, while, apart from the energy range, the current beam size and the flux are not yet compatible with requirements for patient studies, we believe that the expected evolution in compact light source technology will enable *in-vivo* application in the future.

In conclusion, we showed that the compact synchrotron technology offers great potential in the field of coronary angiography by reducing the amount of required contrast media concentration. Quasi-mono-energetic x-ray beams from compact sources pave the way for investigating contrast media with higher atomic number as patients will benefit from dose and contrast media reduction.

## Methods

### Working principle of the MuCLS

**Figure 4:**
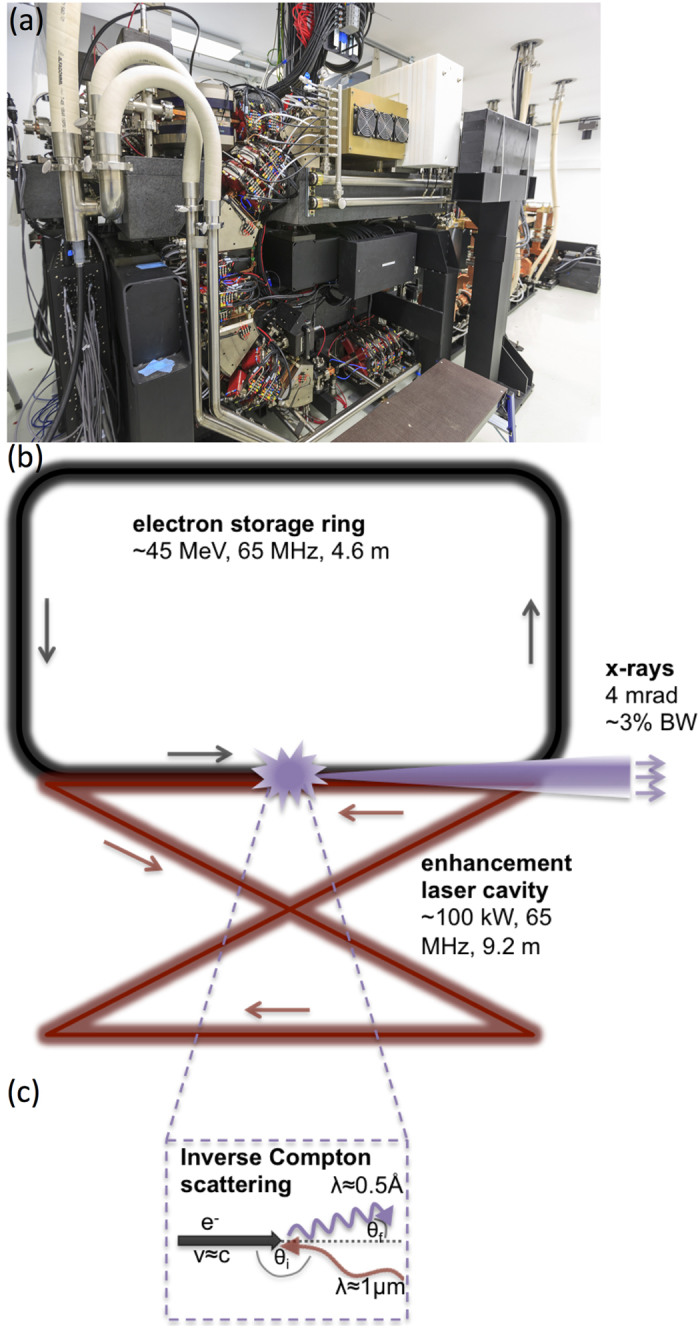
The Munich Compact Light Source (MuCLS). (**a**) Photograph showing the transport line and part of the linear accelerator. (**b**) Schematic drawing of the laser-electron storage ring. (**c**) Diagram of inverse Compton scattering (angles exaggerated).

The MuCLS is a compact synchrotron based on inverse Compton scattering and was developed and manufactured by Lyncean Technologies Inc., USA. As a relativistic electron bunch collides with a laser pulse, x-ray photons with an energy *E*_*x*_ of


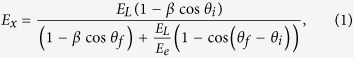


are produced, where *β* = *v*/*c* with *v* and *c* the electron speed and speed of light, respectively, *E*_*x*_ is the x-ray energy, *E*_*L*_ is the laser photon energy, *E*_*e*_ is the electron energy, and *θ*_*i*_ and *θ*_*f*_ are incident and final scattering angles as illustrated in Fig. 4(c)^17^. In good approximation for head-on collision (*θ*_*i*_ = *π*) and backscattering (*θ*_*f*_ = 0, cf. Fig. 4(c)), the equation simplifies to


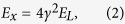


where 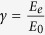 with *E*_0_ the rest energy of the electron. In order to ensure a constantly high x-ray flux, the electron bunch is stored in a miniature storage ring and the laser pulse is stored in a high-finesse optical cavity. Their revolution frequencies are matched so they collide at the interaction point upon each revolution (cf. Fig. 4(a))^1^. In the wave regime, the collision process can be regarded in analogy to a permanent magnet undulator, as the laser pulse is seen as a counter-propagating electromagnetic wave by the electrons. As the undulator period is given by half the laser wavelength and therefore is ~μm instead of ~cm, the electron energy and thus the storage ring circumference can be reduced by a factor of ~200 compared to that of a 3^rd^ generation synchrotron^17^. The produced x-rays are collimated to a 4 mrad cone. The x-ray beam is quasi-monochromatic, partially coherent and the energy is tunable between 15 and 35 keV by adjusting the electron energy. The MuCLS currently offers a flux of up to 1.4 ⋅ 10^10^ photons per second, and a source size of approximately 45 × 45 μm^2^ 2. At a distance of about 16 m from the interaction point, the beam has an elliptic shape of 62 mm × 74 mm, as pictured in Fig. 3(b).

### Simulation

X-ray projection images were simulated using a spectral forward projector. According to the Lambert-Beer law, the projection image is given by


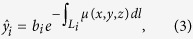


where 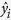 and *b*_*i*_ are the mean number of photons recorded by the detector with and without the sample in place, respectively, and i is an index running over all detector pixels. The line integrals over the spatially varying attenuation coefficient *μ*(*x, y, z*) can be replaced by sums and thus 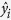 can be rewritten as


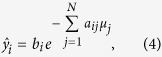


where *a*_*ij*_ is a geometric factor describing how much *μ*_*j*_ contributes to the line integral 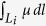. For a polychromatic x-ray beam, this forward projector needs to be extended to include the x-ray spectrum consisting of K energy bins:


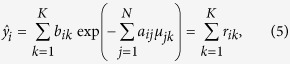


where *b*_*ik*_ is the average number of photons per energy bin *k* in the flatfield and *μ*_*jk*_ is the attenuation coefficient in voxel *j* for the energy bin *k*. For simplification, a perfect flatfield is assumed, i.e. *b*_*i*_ = *b*∀*i*.

The forward model yields the average number of photons behind the object, 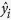. We then calculated the detector projection image *d*_*i*_ for an ideal energy-integrating detector with a 700 µm CsI scintillator:


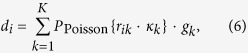


where *k* are the energy bins, *r*_*ik*_ is the number of photons per energy bin behind the sample, and *κ*_*k*_ is the detector quantum efficiency in energy bin *k*. The detector signal *r*_*ik*_ ⋅ *κ*_*k*_ is assumed to be Poisson distributed. This signal is then multiplied by the detector gain *g*_*k*_ for photons in the energy bin *k*, with the gain being proportional to the photon energy, *g*_*k*_ ∝ *E*_*k*_.

### Simulated projections of coronary artery and CNR analysis

A coronary artery was segmented from a CT scan of a human patient. Projection images of the coronary artery embedded in muscle tissue were calculated with the simulation as described above for different spectra and for different contrast agent substances and concentrations within the artery.

The two contrast agents we evaluated were iodine and gadolinium. The parameters for all simulations that were performed are summarized in Table 3. We used attenuation coefficients as provided by NIST^18^. The conventional X-ray tube spectra were simulated based on^19^ for typical clinical settings of acceleration voltage and filtration for a tungsten anode. The CLS spectrum at an energy of 35 keV was measured by an energy-dispersive detector (Amptek X-123, Amptek Inc., USA). For the gadolinium simulation, the measured MuCLS spectrum was rescaled. The CLS and X-ray tube spectra are shown together with the mass-energy attenuation coefficients of iodine and gadolinium in Figs 1(a) and 2(a), respectively. The number of photons in the flatfield was chosen such that the absorbed dose was equal for x-ray tube and CLS spectrum for each contrast agent, and such that the number of transmitted photons was equal for the 60 kVp and the 90 kVp x-ray tube spectra.

In order to account for statistical variations due to Poisson noise, the simulation was performed 10 times for each setting of spectrum and contrast agent substance and concentration to be studied. The standard deviation due to the statistical variation is given with the results. Several regions of interest (ROIs) at different positions in the coronary vascular tree were selected for evaluation of the CNR in order to investigate the contrast between vessel and muscle tissue. The used ROIs are highlighted in Figs 1 and 2 and their size was 8 × 8 and 4 × 4 pixel for thicker and thinner vessels, respectively.

The CNR was calculated according to the definition


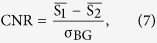


where 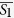 and 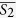 are the average signals in two ROIs which should be compared, and *σ*_*BG*_ is the standard deviation within a larger ROI located in the background region.

### Experimental mono-energetic angiography image

The MuCLS was tuned to 35.0 keV x-ray peak energy, with a flux of approximately 1.1 × 10^6^ photons/s mm^2^ at the sample position. A spectrum of the x-ray beam acquired with an Amptek X-123 detector is shown in Fig. 1(a). An excised porcine heart was placed in a waterbath (photograph shown in Fig. 3(a)) and iodine-based contrast agent (Solutrast 370, Bracco Imaging Deutschland GmbH) was injected into the coronary artery. A flatpanel detector (Varian PaxScan 2520DX, Varian Medical Systems Inc., USA), equipped with a Gd_2_O_2_S scintillator, with a pixel size of 127 μm was placed at 16.5 m from the x-ray source point. The sample-detector distance was 80 cm, corresponding to an effective pixel size of 121 μm. An image was acquired with an exposure time of 1 second and flatfield-corrected.

## Additional Information

**How to cite this article:** Eggl, E. *et al*. Mono-Energy Coronary Angiography with a Compact Synchrotron Source. *Sci. Rep.* **7**, 42211; doi: 10.1038/srep42211 (2017).

**Publisher's note:** Springer Nature remains neutral with regard to jurisdictional claims in published maps and institutional affiliations.

## Figures and Tables

**Table 1:** CNR calculated from simulated projections for two different concentrations of iodine-based contrast media.

ROI	60 kVp	35 keV	gain	60 kVp	35 keV	gain
	75 mg/ml iodine			50 mg/ml iodine		
1	8.51 ± 0.49	9.96 ± 0.36	17%	5.80 ± 0.32	7.18 ± 0.26	24%
2	3.24 ± 0.34	3.79 ± 0.19	17%	2.26 ± 0.20	2.83 ± 0.23	25%
3	6.33 ± 0.34	7.67 ± 0.38	21%	4.22 ± 0.21	5.41 ± 0.12	28%
4	7.49 ± 0.44	9.10 ± 0.47	22%	5.14 ± 0.21	6.54 ± 0.27	27%
5	6.42 ± 0.35	7.81 ± 0.22	22%	4.43 ± 0.17	5.49 ± 0.23	24%
6	39.54 ± 1.85	29.13 ± 1.07	−26%	34.40 ± 1.17	27.62 ± 0.61	−20%

The standard deviation from the statistical variation of the simulation is given with the mean value of the CNR of the 10 simulation runs.

**Table 2:** CNR calculated from simulated projections for two different concentrations of gadolinium-based contrast media.

ROI	90 kVp	55 keV	gain	90 kVp	55 keV	gain
	75 mg/ml gadolinium			50 mg/ml gadolinium		
1	5.27 ± 0.18	7.42 ± 0.32	41%	3.55 ± 0.17	5.01 ± 0.13	41%
2	1.78 ± 0.41	2.87 ± 0.28	62%	1.27 ± 0.30	1.65 ± 0.22	30%
3	3.91 ± 0.15	5.57 ± 0.20	43%	2.40 ± 0.17	3.62 ± 0.18	51%
4	4.75 ± 0.15	6.75 ± 0.27	42%	3.04 ± 0.21	4.53 ± 0.29	49%
5	3.93 ± 0.17	5.69 ± 0.31	45%	2.64 ± 0.20	3.77 ± 0.16	43%
6	30.19 ± 0.95	33.83 ± 0.89	12%	24.93 ± 0.55	29.45 ± 0.87	18%

The standard deviation from the statistical variation of the simulation is given with the mean value of the CNR of the 10 simulation runs.

**Table 3:** Simulation parameters.

Contrast Agent	Iodine	Gadolinium
K-edge energy	33.17 keV	50.24 keV
X-ray tube	60 kVp, Tungsten 0.7 mm Cu filter	90 kVp, Tungsten 0.7 mm Cu filter
MuCLS	35.0 keV peak energy 4% FWHM	55.0 keV peak energy 4% FWHM
Contrast media concentrations	high: 75 mg/mllow: 50 mg/ml	high: 75 mg/mllow: 50 mg/ml
Photons in Flatfield (x-ray tube/MuCLS)	4230/3000	15000/15000
Detector sensor	700 *μm* CsI	700 *μm* CsI
QE of detector sensor (x-ray tube/MuCLS)	98.3%/99.5%	88.2%/97.27%
Muscle tissue depth	3 cm	10 cm
